# Relationship between segmental trunk control and gross motor development in typically developing infants aged from 4 to 12 months: a pilot study

**DOI:** 10.1186/s12887-019-1791-1

**Published:** 2019-11-11

**Authors:** Tamis W. Pin, Penelope B. Butler, Hon-Ming Cheung, Sandra Lai-Fong Shum

**Affiliations:** 1 Department of Rehabilitation Sciences, The Hong Kong Polytechnic University, Hung Hom, Kowloon, Hong Kong; 2 Health, Exercise and Active Living, Manchester Metropolitan University, Manchester, UK; 3 Department of Paediatrics, Prince of Wales Hospital, Shatin, Hong Kong; 4 Physiotherapy Department, Prince of Wales Hospital, Shatin, Hong Kong

**Keywords:** Postural control, Longitudinal, Infants, Gross motor skills

## Abstract

**Background:**

Trunk control is generally considered to be related to gross motor development. However, this assumption has not been validated with clinical data. This pilot study was the first of its kind to examine the longitudinal development of segmental trunk control and gross motor development from 4 to 12 months of age in typically developing full-term infants.

**Methods:**

A convenience cohort of 20 healthy full-term infants (mean gestation = 39.0 weeks, SD 1.2; mean birthweight = 2975.0 g, SD 297.0; males = 10) was recruited. All study infants were tested and scored monthly by independent assessors using the Segmental Assessment of Trunk Control and the Alberta Infant Motor Scale from 4 to 12 months of age.

**Results:**

A developmental trend of segmental trunk control was found in the infants. Static vertical upright trunk control developed prior to active and reactive control. Statistically significant correlations were found between trunk control status and gross motor development mainly in prone and sitting positions from 8 months of age onwards (all *p* < 0.004, Spearman’s r ranged from 0.644 to 0.798).

**Conclusions:**

This pilot study provides preliminary clinical evidence to support the inter-dependency between vertical upright trunk control and gross motor development in young infants, particularly as upright functional skills are gained. This suggests that a dual focus on training upright trunk control alongside gross motor skills could be of benefit in the treatment of infants with movement disorders.

## Background

Efficient trunk control allows an individual to function in an upright vertical posture without losing balance [1]. Trunk control plays a significant role in motor development when a typically developing (TD) infant starts to move against the gravity during their first 12 months of life but is commonly delayed in infants and young children with movement disorders [1].

In clinical settings, assessment of trunk control status in infants and young children is commonly made by simple observation during a developmental assessment [1]. More specific outcome measures have recently been developed to assess trunk control directly, such as the Trunk Control Measurement Scale [2], or to assess balance, such as the Pediatric Reach test [3], with the latter assuming that if an individual is able to maintain balance at rest or during movements, then efficient trunk control is present [4]. These assessments are commonly constrained by considering the trunk as one unit despite its multi-segmental composition [5]. It has already been shown that the developmental changes in maintaining upright stability are specific to different regions of the trunk in TD young infants before they acquire independent sitting [6]. Studies have further shown that trunk control is developed in a segmental cephalic to caudal sequence in TD young infants [7] and before they have achieved independent sitting, their reaching performance is highly correlated with the level of their segmental trunk control [8, 9].

To date, the relationship between segmental trunk control and gross motor development in TD infants from a developmental perspective is unknown: for example, does an infant require upper, middle or lower thoracic control in order to prop on extended arms in prone or is propping not related to thoracic control? Is independent sitting related to acquisition of lumbar control? Conventional outcome measures of developmental milestones and trunk control do not, and cannot, examine this relationship between segmental trunk control and gross motor development. However, this information is particularly important for clinicians working with infants and young children with movement disorders. Curtis and colleagues have already proven a positive association between segmental trunk control and gross motor function in children with cerebral palsy [10]. Adequate support at the appropriate trunk segment seemed to facilitate better sitting posture, movement quality and hand function in young children with movement disorders [11]. Knowledge of the correlation between segmental trunk control and typical gross motor development is likely to enhance our understanding of how to assess and to enhance functional abilities in infants and young children with movement disorders.

The objectives of the present study were to (i) systematically investigate and document the development of segmental trunk control in TD full-term infants from 4 to 12 months of age and (ii) examine if any correlation existed between segmental trunk control acquisition and gross motor development in prone, supine, sitting and standing positions. Although the present study was part of a series examining the relationship between segmental trunk control and gross motor development in young infants, infants were recruited to this study specifically to investigate these objectives.

## Methods

Twenty full-term infants were recruited by convenience via personal contact. The inclusion criteria were: (1) born at or after 37 weeks of gestation and (2) no adverse pre-, peri-, or postnatal histories as reported by their parents or family doctor. The exclusion criteria were infants with known congenital abnormalities or genetic syndromes. Ethical approval was granted from the first author’s affiliation. Informed consent was signed by all parents prior to data collection.

All study infants were assessed monthly from 4 to 12 months of age at their home, i.e. 9 visits per infant. The Segmental Assessment of Trunk Control (SATCo) [12] was used to assess their neutral vertical trunk control status in a seated position. The SATCo considers the head and neck as one segment and sub-divides the trunk into five further segments. Head/trunk control is dichotomously credited segment by segment if a vertical upright sitting posture can be maintained under three conditions: at rest (static control), during head and/or arm movements (active control) and after external perturbations (reactive control) [12]. The SATCo identifies at which head/trunk segment control is currently being learnt for each of static, active and reactive control. It is one of the rare outcome measures to assess all three types of trunk control [13] and its reliability and validity on infants and young children has recently been established [14]. The study infants were also tested using the Alberta Infant Motor Scale (AIMS) [15] to establish gross motor development. The AIMS is a norm-referenced standardised assessment of gross motor skills for infants from birth until 18 months of age or until the infants achieve independent walking.

All the testing was conducted by the first author (TWP). The infants’ performance during both SATCo and AIMS was captured from front-oblique and side views using two cameras. A similar set-up was successfully used in our previous studies using the SATCo on young infants [14, 16]. Both tests were scored according to the published criteria from the video-recordings [12, 15]. TWP scored all the AIMS and her reliability in using the AIMS on infants has been previously proven [17]. An independent assessor (second author, PBB) scored each SATCo from the videos of both views: PBB is the author of the SATCo and has proven reliability in using the test [12].

In general, sample size calculation is not necessary for pilot studies [18]. As there is no previous study examining the relationship between trunk control status and gross motor development in typically developing infants, a sample size of 20 infants was considered appropriate for statistical analyses of the present study.

The SATCo is an ordinal scale and thus a number 1 to 7 was allocated to each segment to enable statistical analysis with 1 for head control, 2 for upper thoracic, 3 for mid-thoracic, 4 for lower thoracic, 5 for upper lumbar, 6 for lower lumbar and 7 for learning full trunk control; 8 was used for full gained trunk control. The distinction between scores 7 and 8 is that a score of 7 indicates that the infant had not fully mastered full trunk control to enable sitting independently without hand support. A score of 8 indicates that full trunk control was present and the infant able to sit independently. This format has been used in previous studies of the SATCo [12, 14]. At each test, an infant would have 3 numerical values to indicate the respective segmental levels of static, active and reactive control learning. For example, scores of 6, 4 and 3 denotes that an infant is currently learning static trunk control at the lower lumbar segment, active control at upper lumbar, and reactive control at mid thoracic. The AIMS test items are sub-divided into four positions of prone, supine, sitting and standing. Each test item represents a gross motor skill commonly observed in TD infants. A point is credited for each observed motor skill, resulting a sub-score in each position. The sum of these four sub-scores is the total score [15].

Friedman test [19] was used to examine the developmental trend (changes over time) of the SATCo from 4 to 12 months of age. The Friedman test is a non-parametric counterpart of repeated measures of ANOVA taking time as a repeated factor and thus controlling for the repeated measurements over time. As we were interested in the relationships between the three different conditions of segmental trunk control (i.e. static, active and reactive) and gross motor development, we examined the relationship of each condition of trunk control separately. Specifically, the SATCo scores and the four sub-scores of the AIMS (i.e. gross motor development in each of the four positions) were used to test the strength of correlations between trunk control status and gross motor development using the Spearman’s correlation (r) [19]. If r ≤ 0.25, it represents little or no correlation and fair correlation if *r* = 0.25 to 0.50. It is considered as moderate to good correlation if *r* = 0.50 to 0.75, and good to excellent if ≥0.75 [19]. All statistical significance levels were adjusted for the repeated measures at *p* = 0.004 (based on 3 aspects of segmental trunk control and 4 AIMS sub-scores). In order to obtain adequate data points to examine the developmental trends of both trunk control status and gross motor development in the infants, infants should be examined at a minimum of 80% of these 9 visits.

## Results

Twenty-one infants were recruited and assessed between November 2014 and March 2018. Data of one infant was discarded as she had severe stranger anxiety with constant crying and no engagement with any of the test procedures during the first or second session. The present results, therefore, were based on 20 infants (mean gestatio*n* = 39.0 weeks, SD 1.2; mean birthweight = 2975.0 g, SD 297.0; *n* = 10 males, 50%). Five (2.8%) data points were missing (*n* = 3 families unable to schedule and *n* = 2 unable to test due to infant distress).

**Fig. 1:**
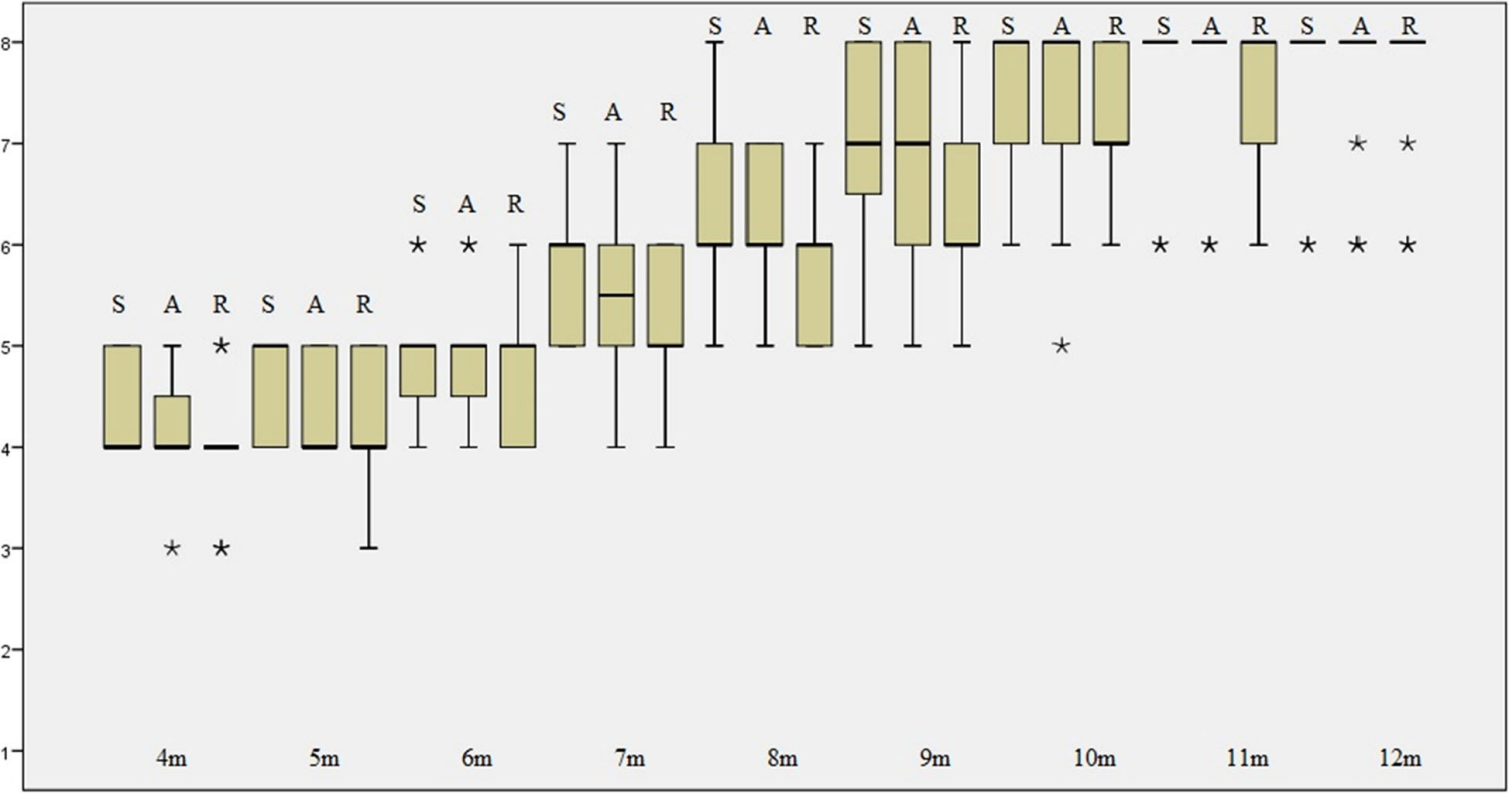
Developmental trends of segmental trunk control from 4 to 12 months in study infants. S- static control, A- active control, R- reactive control. Numbers on the y-axis are the SATCo trunk segmental level at which control was being learnt (1 = head control, 2 = upper thoracic level, 3 = mid-thoracic, 4 = lower thoracic, 5 = upper lumbar, 6 = lower lumber, 7 = full trunk control, and 8 = full trunk control achieved). The solid line represents the medians of the group at each age group. The boxes and the whiskers represent the spread of the data within that age group. The asterisks represent outliers in that age group. Please note that the SATCo is an ordinal scale and the non-integral numbers reported in the figure were purely for statistical purposes. In real life situations, no half-level would be credited to the infants

The static, active and reactive development of segmental trunk control of the infants showed a significant time effect (Chi-square χ = 117.22, 116.82, and 117.32 respectively, all *p* < 0.001) (Fig. 1).

Significant correlations were found between the SATCo and AIMS scores mainly seen from 8 months of age onwards (Table 1). Significant correlations were found between the SATCo static and active scores, and sit sub-scores of the AIMS at 8, 10 and 11 months. The static and active SATCo scores at 10 months, and reactive SATCo score at 11 months was significantly correlated with the AIMS prone sub-scores at the corresponding ages. The reactive SATCo score was also significantly correlated with the AIMS sit sub-scores at 11 months.

**Table 1:** Spearman’s correlations between SATCo scores and AIMS sub-scores

SATCo median score (range)	AIMS prone sub-score (max = 21)	AIMS supine sub-score (max = 9)	AIMS sit sub-score (max = 12)	AIMS stand sub-score (max = 16)
*4 months*	4.11 (0.88)	4.47 (1.35)	1.68 (0.67)	1.95 (0.23)
Static	4 (4 to 5)			
r (*p*)	0.022 (*p* = 0.928)	0.162 (*p* = 0.508)	0.396 (*p* = 0.093)	0.180 (*p* = 0.461)
Active	4 (3 to 5)			
r (*p*)	0.134 (*p* = 0.585)	0.316 (*p* = 0.187)	0.390 (*p* = 0.099)	0.127 (*p* = 0.605)
Reactive	4 (3 to 5)			
r (*p*)	−0.131 (*p* = 0.593)	0.026 (*p* = 0.915)	0.120 (*p* = 0.623)	− 0.028 (*p* = 0.910)
*5 months*	5.80 (1.06)	5.35 (1.90)	2.60 (1.05)	2.05 (0.22)
Static	5 (4 to 5)			
r (*p*)	0.272 (*p* = 0.246)	0.376 (*p* = 0.102)	0.517 (*p* = 0.020)	0.150 (*p* = 0.527)
Active	4 (4 to 5)			
r (*p*)	0.344 (*p* = 0.138)	0.337 (*p* = 0.146)	0.329 (*p* = 0.156)	0.254 (*p* = 0.281)
Reactive	4 (3 to 5)			
r (*p*)	0.479 (*p* = 0.033)	0.308 (*p* = 0.186)	0.169 (*p* = 0.475)	0.300 (*p* = 0.199)
*6 months*	7.60 (2.04)	6.30 (2.08)	4.10 (1.71)	2.30 (0.47)
Static	5 (4 to 6)			
r (*p*)	0.268 (*p* = 0.254)	−0.092 (*p* = 0.699)	0.178 (*p* = 0.453)	0.329 (*p* = 0.157)
Active	5 (4 to 6)			
r (*p*)	0.268 (*p* = 0.254)	- 0.092 (*p* = 0.699)	0.178 (*p* = 0.453)	0.329 (*p* = 0.157)
Reactive	5 (4 to 6)			
r (*p*)	0.218 (*p* = 0.356)	−0.268 (*p* = 0.254)	0.218 (*p* = 0.356)	0.217 (*p* = 0.357)
*7 months*	9.84 (3.78)	7.32 (2.08)	6.74 (2.77)	2.74 (0.45)
Static	6 (5 to 7)			
r (*p*)	0.436 (*p* = 0.062)	0.023 (*p* = 0.925)	0.507 (*p* = 0.027)	0.075 (*p* = 0.760)
Active	5 (4 to 7)			
r (*p*)	0.302 (*p* = 0.209)	0.180 (*p* = 0.461)	0.440 (*p* = 0.059)	−0.132 (*p* = 0.589)
Reactive	5 (4 to 6)			
r (*p*)	0.308 (*p* = 0.199)	−0.068 (*p* = 0.781)	0.492 (*p* = 0.033)	0.026 (*p* = 0.917)
*8 months*	11.60 (4.08)	8.45 (1.15)	8.60 (2.46)	3.25 (0.79)
Static	6 (5 to 8)			
r (*p*)	0.549 (*p* = 0.012)	0.544 (*p* = 0.013)	0.674 (*p* = 0.001)*	0.575 (*p* = 0.008)
Active	6 (5 to 7)			
r (*p*)	0.591 (*p* = 0.006)	0.653 (*p* = 0.002)*	0.644 (*p* = 0.002)*	0.538 (*p* = 0.014)
Reactive	6 (5 to 7)			
r (*p*)	0.562 (*p* = 0.010)	0.440 (*p* = 0.052)	0.464 (*p* = 0.039)	0.372 (*p* = 0.107)
*9 months*	15.25 (3.61)	8.65 (0.59)	10.40 (2.14)	5.10 (2.00)
Static	7 (5 to 8)			
r (*p*)	0.523 (*p* = 0.018)	0.054 (*p* = 0.820)	0.543 (*p* = 0.013)	0.351 (*p* = 0.129)
Active	7 (5 to 8)			
r (*p*)	0.486 (*p* = 0.030)	0.242 (*p* = 0.303)	0.455 (*p* = 0.044)	0.332 (*p* = 0.152)
Reactive	6 (5 to 8)			
r (*p*)	0.551 (*p* = 0.012)	0.123 (*p* = 0.606)	0.529 (*p* = 0.017)	0.399 (*p* = 0.081)
*10 months*	17.30 (2.64)	8.75 (0.44)	11.40 (1.31)	7.10 (2.73)
Static	8 (6 to 8)			
r (*p*)	0.739 (*p* < 0.001)*	0.268 (*p* = 0.267)	0.798 (*p* < 0.001)*	0.607 (*p* = 0.006)
Active	8 (5 to 8)			
r (*p*)	0.764 (*p* < 0.001)*	0.383 (*p* = 0.106)	0.654 (*p* = 0.002)*	0.461 (*p* = 0.047)
Reactive	7 (6 to 8)			
r (*p*)	0.426 (*p* = 0.069)	0.359 (*p* = 0.131)	0.612 (*p* = 0.005)	0.351 (*p* = 0.140)
*11 months*	18.26 (3.03)	8.68 (0.48)	11.63 (0.96)	8.42 (2.39)
Static	8 (6 to 8)			
r (*p*)	0.547 (*p* = 0.015)	0.505 (*p* = 0.027)	0.765 (*p* < 0.001)*	0.539 (*p* = 0.017)
Active	8 (6 to 8)			
r (*p*)	0.547 (*p* = 0.015)	0.505 (*p* = 0.027)	0.765 (*p* < 0.001)*	0.539 (*p* = 0.017)
Reactive	8 (6 to 8)			
r (*p*)	0.645 (*p* = 0.003)*	0.642 (*p* = 0.003)*	0.778 (*p* < 0.001)*	0.284 (*p* = 0.239)
*12 months*	19.37 (1.38)	8.53 (0.51)	11.84 (0.69)	10.32 (2.69)
Static	8 (6 to 8)			
r (*p*)	0.546 (*p* = 0.016)	0.362 (*p* = 0.128)	0.687 (*p* = 0.001)*	0.542 (*p* = 0.017)
Active	8 (6 to 8)			
r (*p*)	0.614 (*p* = 0.005)	0.197 (*p* = 0.419)	0.576 (*p* = 0.010)	0.610 (*p* = 0.006)
Reactive	8 (6 to 8)			
r (*p*)	0.614 (*p* = 0.005)	0.197 (*p* = 0.419)	0.576 (*p* = 0.010)	0.610 (*p* = 0.006)

Numbers in brackets are standard deviations unless specified

*AIMS* Alberta Infant Motor Scale, *r* Spearman’s correlation, *SATCo* Segmental Assessment of Trunk Control

*significant at *p* = 0.004

**Fig. 2:**
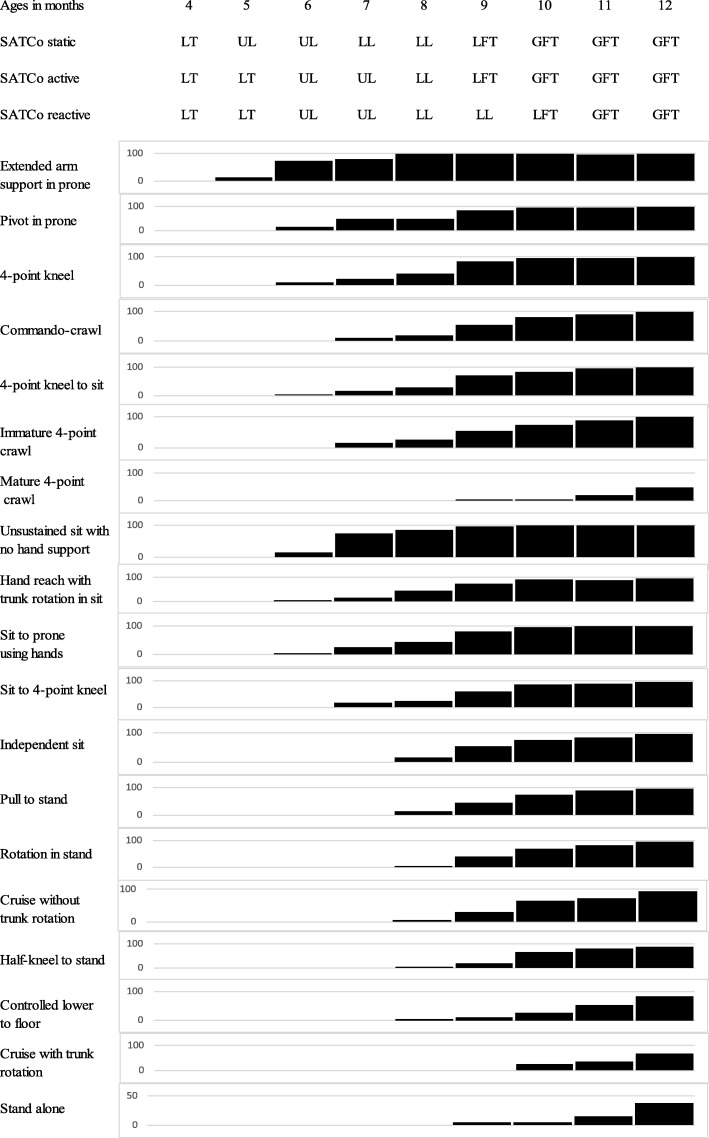
Segmental trunk control status and gross motor function from 4 to 12 months in study infants. LT = lower thoracic, UL = upper lumbar, LL = lower lumber, LFT = learning full trunk control, GFT = gained full trunk control

At 7 months, over two-thirds (79%) of the infants were able to prop on extended arms in prone but only one-fifth (21%) of them were getting onto four-point kneeling and about 13% started to crawl on stomach or on hands and knees. Over two-thirds (74%) of the infants had just started to sit without hand support but less than one-fifth (16%) had achieved independent sitting. The level of learning static control at this 7-month age was at the lower lumbar segment (Fig. 2).

At 8 months, moderate to good correlation was found between static and active segmental trunk control status and gross motor skills of the study infants in the sitting position (all *r* = 0.644 to 0.674, Table 1). The levels for learning both static and active control were lower lumbar to full trunk but upper to lower lumbar for reactive control (Table 1 and Fig. 2). In prone, an increasing number of infants were using four-point kneeling (40%), crawling on stomach (20%), moving in and out of four-point kneeling (30%) and early crawling on hands and knees (25%). Forty-five percent of the infants were sitting independently and the same percentage starting to move from sitting to prone but mainly using arms to do so.

At 9 months, the levels for learning static and active control were at full trunk and eight infants (40%) had gained full trunk control. The learning level for reactive control was lower lumbar to full trunk. Over half of the infants either crawled on stomach (55%) or on hands and knees (55%) and 70% of infants moved in and out of four-point kneeling. Most of the infants sat independently with trunk rotation shown (75%) and could move from sitting to prone either mainly using their arms (80%) or moving from sitting to four-point kneeling position (60%). Over half of the infants (55%) were very mobile in unaided sitting and moved in and out of sitting easily (Fig. 2).

At 10 months of age, the infants continued to consolidate their full trunk control and motor skills in the four-point kneeling and sitting positions. This was reflected in the excellent correlation between the SATCo static and active scores and the AIMS prone and sit sub-scores (all *r* > 0.65, Table 1). Most of the infants were able to move in and out of sitting to four-point kneeling positions easily and crawl in an immature pattern, i.e. hips abducted and increased lumbar lordosis. At this stage, over two-thirds of the infants were able to pull to stand (75%) and stand with support (70%) and two-thirds (65%) of the infants cruised along furniture and pulled to stand via half-kneeling. One-quarter (25%) of the infants could lower themselves from standing to the floor with control and cruise with trunk rotation shown (Fig. 2).

By 11 months of age, most of the infants (89%) had gained full static and active trunk control and 74% had gained full reactive trunk control. Good to excellent correlations were found between trunk control status and motor skills in sitting positions (r ranged from 0.765 to 0.778, Table 1) and moderate correlation between reactive trunk control and motor skills in the prone position (*r* = 0.645). Twenty-one percent of the infants crawled on hands and knees with good trunk alignment and trunk rotation. Half of the infants were able to lower from standing to the floor with control (53%) while 37% cruised along furniture with trunk rotation. In contrast, the two infants who had not gained full static and active trunk control by this time, scored low in the prone, sit and stand sub-scores (Fig. 2).

At 12 months, 89% of study infants had gained full static, active and reactive trunk control. Moderate to good correlation was found between the static trunk control status and motor skills in sitting position (*r* = 0.687, Table 1). Nearly half of the study infants crawled on hands and knees with good alignment and trunk rotation (47%) and all except one infant sat independently and moved in and out of sitting easily (95%). One-third of the infants (32%) started to walk unaided at this age. Following from 11 months of age, the two infants who had not gained full static, active and reactive trunk control had the lowest prone, sit and stand sub-scores and consequently the lowest total score.

Thirty-four of the AIMS total scores across the study period were under the recommended cut-off 5th percentile [20] (*n* = 5, 7, 6, 3, 2, 4, 4, 2 and 1 at 4, 5, 6, 7, 8, 9, 10, 11 and 12 months respectively). These low scores were predominantly from two infants (6 out 9 testings of Infant 005 and all testings of Infant 016).

## Discussion

The development of trunk control is considered to play a significant role in gross motor development in infants. However, trunk control status is not routinely assessed for infants per se as it is presumed that if the infant can achieve some motor milestones, e.g. independent sitting, the infant has sufficient trunk control [21]. Our present pilot study addressed this situation by investigating segmental trunk control development in typically developing infants from 4 to 12 months of age and exploring the correlation with their gross motor development.

The developmental trend of neutral vertical segmental trunk control was shown to be cephalic to caudal (Fig. 1), which accords with the traditional cephalic-to-caudal theory of motor control [22] and with previous findings [9, 14]. Infants sequentially developed full static and active trunk control by 10 months and reactive control by 11 months as shown in the plateau of the respective SATCo scores after 10 and 11 months (Table 1, Figs. 1 and 2). It is apparent that there are time differences among the three types of trunk control acquired, with static control preceding active and then reactive control (Fig. 1). This coincides with the general concept of that static postural control develops prior to dynamic postural control (active and reactive control) [22].

The SATCo assesses upright vertical head/trunk control. This posture is biomechanically the most challenging since it requires full control of all unsupported segments to maintain the upright position [23]. The SATCo tests this neuromuscular control under static, active and reactive conditions, introducing management of both gravitational and external challenge [23]. This contrasts with non-vertical postures such as lying where the control demands are greatly simplified and there is no necessity to actively maintain dynamic stability of a column of unsupported segments [12]. This may not be true of infants with neurological disorders and the addition of appropriate external support to reduce the control demands may then prove beneficial [5, 11, 23]. It is interesting to note that reaching ability has been related to segmental development of trunk control in TD infants [8, 9] and preterm infants [24] with acquisition of lumbar control improving the quality of reaching ability. Biomechanically, lumbar control will confer trunk stability for effective arm function while also enabling hands-free sitting [25].

Our discussion on the correlation between the trunk control status and gross motor development focusses on the gross motor skills in each of the four testing positions in the AIMS, rather than referring to the total AIMS score. The non-significant correlations between the SATCo and AIMS scores at early ages, i.e. 4 to 7 months, were expected as most of the infants were developing gross motor skills in reclined positions of supine and prone (Table 1). Infants below the age of 7 months are gaining gross motor skills but these skills do not generally use the neutral vertical posture. As an example, infants are learning to roll and they gain independent sitting in stages, first using hand support with forward lean of the trunk [26]. However these gross motor skills do not use a neutral vertical trunk posture. Infants’ need for control of a vertical upright trunk was, therefore, low when compared to upright sitting or standing positions. The SATCo tests only the segmental development of neutral vertical postural control of the trunk. This may help to explain the general lack of correlation before 8 months but further testing with larger numbers of infants would be valuable. From 8 months of age, correlations began to emerge between SATCo and AIMS: infants were gaining static, active and reactive control at the lower lumbar segment and using more upright postural gross motor skills such as transferring in and out of sitting. These more active functional abilities will necessitate greater control of the trunk, particularly in the vertical upright posture of sitting. Infants were also transitioning between crawling on stomach and crawling on hands and knees. Crawling is not a truly ‘vertical posture gross motor skill’ but it may start to address the question, posed in the Introduction, about the association between gross motor function and segmental vertical trunk control, giving valuable information for therapists.

Two infants (Infants 005 and 016) had not gained full static and active trunk control by 11 to 12 months and had low scores in the prone, sit and stand sub-scores of the AIMS. Their total AIMS scores were under 5th percentile of the AIMS norms, implying that they might be at risk of motor delay, especially Infant 016 [20]. Their level of learning static and active control was at lower lumbar to full trunk. This lends further support to the close relationship and inter-dependence between segmental trunk control and gross motor function in infants.

The present results have provided further support to our previous investigation of segmental trunk control development and its correlation with gross motor function in infants born before 30 weeks of gestation (currently under review for publication). Our study has provided preliminary evidence that segmental trunk control and gross motor development are inter-dependent using clinical data. We believe that this relationship between trunk control and gross motor skills should be borne in mind by clinicians treating infants and young children with movement disorders. The addition of simultaneous training of upright trunk control when training gross motor skills could be advantageous and worthy of future research.

The present results must be considered in light of methodological limitations. Although this was a pilot study of 20 infants, this sample size remains small when compared with the number of infants born each year. Nevertheless, both significant and non-significant correlations are equally revealing in terms of increasing understanding of the relationship between segmental trunk control and motor function in TD infants, especially in very young infants of 4 to 7 months. The reader should be aware that those infants who scored below the cut-off 5th percentile of the AIMS, i.e. infants at risk of motor delay, were included for statistical analyses. This inclusion allows the study sample to reflect the full spectrum of gross motor abilities and trunk control seen among the TD infant population. The independent scorings of segmental trunk control of the study infants was a strength of this pilot study.

## Conclusions

We have demonstrated preliminary evidence on the longitudinal development of segmental trunk control in TD infants from 4 to 12 months of age, indicating a developmental trend of segmental trunk control with upright static control developing prior to active and reactive control. Based on the statistically significant correlations between vertical trunk control status and acquisition of gross motor milestones starting from 8 months of age, trunk control and gross motor performance were closely related. Hence, we recommend simultaneous training of upright trunk control and task-specific gross motor skills for infants with movement disorders.

## Data Availability

The datasets used and/or analysed during the current study are available from the corresponding author on reasonable request.

## Abbreviations

AIMS: Alberta Infant Motor Scale
SATCo: Segmental Assessment of Trunk Control
TD: Typically developing
